# Development and validation of a screening model for dysphagia in the elderly based on acoustic features

**DOI:** 10.3389/fmed.2025.1719174

**Published:** 2025-12-08

**Authors:** Hongdan Song, Dan Li, Tao Liu, Wei Luo, Xu Dong, Xiaoyan Jin, Shaomei Shang

**Affiliations:** School of Nursing, Peking University, Beijing, China

**Keywords:** dysphagia, XGBoost, acoustic analysis, the elderly, screening

## Abstract

**Background:**

Dysphagia is a prevalent and serious condition among the elderly, yet scalable screening tools are lacking. This study aimed to develop and validate an automated machine learning model based on acoustic features for screening dysphagia risk in the elderly.

**Methods:**

Adhering to TRIPOD guidelines, we conducted a study in three stages: variable screening, model construction, and evaluation. Audio data (voice, cough, swallow) were collected from the elderly in nursing homes. A modeling dataset (Beijing area, *n* = 419) was used to screen key features via LASSO regression. Models were built using Logistic Regression, Random Forest, SVM, and XGBoost, with performance evaluated on an internal test set. The best-performing model was subsequently validated on an external dataset (Shijiazhuang area, *n* = 216).

**Results:**

The XGBoost model demonstrated superior performance, with an area under the curve (AUC) of 0.86 in internal validation and an AUC of 0.71 in external validation, showing good discrimination, calibration, and clinical utility.

**Conclusion:**

The acoustic feature-based XGBoost model serves as an effective and automated tool for screening dysphagia risk in the elderly. It has the potential to assist healthcare professionals in identifying high-risk individuals for early intervention, thereby improving clinical outcomes.

## 1 Introduction

Dysphagia, also known as swallowing disorders or deglutition disorders, refers to a condition where the structure and/or function of organs such as the lower jaw, lips, tongue, soft palate, pharynx, and esophagus are impaired (1). The symptoms of being unable to safely and effectively transport food into the stomach can cause serious complications such as malnutrition, dehydration, and aspiration pneumonia (1), and significantly increase the psychological burden of patients (2, 3) and the pressure on the medical system (3–6). The prevalence rate of dysphagia among the elderly worldwide is as high as 48.1% (7). In China, the prevalence of this disease shows significant differences: it is 13.9% among the elderly in the community, rises to 26.4% in elderly care institutions, while it is as high as 57.7% among the hospitalized elderly (8, 9). Dysphagia screening refers to the initial determination of the existence and severity of dysphagia by identifying a series of related symptoms and signs, which is helpful for the early identification of related problems (10). Numerous studies at home and abroad, expert consensus and clinical guidelines have shown that implementing early assessment and intervention for the population at risk of dysphagia can effectively prevent related complications, control the progression of dysphagia, reduce mortality, and significantly improve prognosis (11–15). Therefore, early dysphagia screening is the key to reducing the burden of dysphagia (10).

The change of swallowing acoustic signal can directly reflect the abnormal swallowing function. Normal swallowing produces a characteristic acoustic signature, typically comprising three distinct components corresponding to specific physiological activities: the initial sound related to laryngeal elevation and epiglottic movement, the main click associated with the opening of the upper esophageal sphincter, and the final sound linked to the separation of the tongue from the pharyngeal wall and the descent of the larynx (16). Specifically, when these acoustic features are abnormal, it indicates the corresponding physiological dysfunction. Prolonged duration may signify a delayed swallowing reflex or inefficient pharyngeal contraction, while abnormal signal properties (e.g., skewness, kurtosis) can indicate irregular bolus flow or the presence of pharyngeal residue (17–21). Furthermore, the presence of pathological adventitious sounds, such as post-swallow wet voice or cough, is a critical acoustic marker of impaired airway protection and potential aspiration, often resulting from incomplete laryngeal closure or weakened pharyngeal motility (22–24). Therefore, as a simple and non-invasive method, swallowing acoustic signal analysis can provide key physiological information reflecting the dynamic process of swallowing and is of significant value in the screening of dysphagia.

In recent years, swallowing acoustic analysis technology has become a research hotspot in the screening of dysphagia due to its non-invasiveness, portability and dynamic monitoring ability (17, 20). The acoustic analysis model based on machine learning has demonstrated high recognition performance in small-sample studies, confirming the representational potential of acoustic features for swallowing function (25–27). However, at present, there is still a lack of a simple, safe, effective and user-friendly screening tool (10, 28). Studies show that many elderly people in communities and elderly care institutions do not routinely undergo dysphagia screening (29), and the lack of effective tools for swallowing function assessment is the main reason (30, 31). The “European Society for Dysphagia - EU Geriatrics White Paper” (10) proposes that the ideal dysphagia screening tool should be suitable for use by caregivers and primary health care providers, and have the characteristics of simplicity, safety, rapidity, accuracy and economy. Due to the degenerative changes of the swallowing organs and the high incidence of latent aspiration in the elderly population, there is an urgent need for a screening tool that is applicable to various environments and has higher safety and accuracy in order to carry out effective intervention and management.

This study constructed a prediction model based on the swallowing acoustic data of the elderly in Beijing, China, and screened out the optimal model through internal verification. Further external verification was conducted using the independent dataset in Shijiazhuang area, confirming that the model has good generalization ability. The research results show that this model demonstrates high clinical practical value among the elderly population with different risk levels. It can provide reliable decision support for clinical risk screening and remote home care, which is significant for the early identification and intervention of dysphagia in the elderly.

## 2 Materials and methods

**FIGURE 1:**
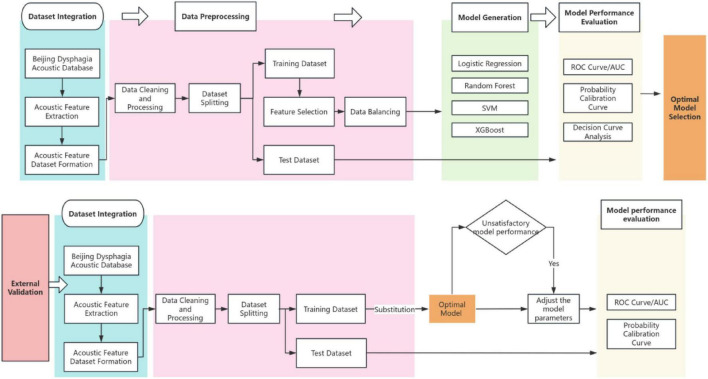
Model construction and validation process.

This study consists of three consecutive steps: ① Screening of identification variables; ② Construction of the recognition model; ③ Evaluation of the recognition model (32, 33). Refer to Figure 1 for a detailed description of the study design.

### 2.1 Study participants

This study took the elderly in nursing homes as the research subjects. During the two time periods from September 2023 to February 2024 and from October 2024 to November 2024, research subjects were recruited from a total of 13 elderly care institutions in Beijing and Shijiazhuang, respectively according to the inclusion and exclusion standards.

Inclusion criteria: ①Age ≥ 60 years old; ② The condition is stable and the patient can eat orally. ③ Voluntarily participate in the research and sign the informed consent form. Exclusion criteria: ① Cognitive or behavioral disorders affect the execution of instructions; ② Acute stage/terminal stage of the disease; ③ Those who are unable to cooperate with the assessment due to other serious physical or mental illnesses.

Based on previous studies, the sample size of this research was determined by taking into account the following factors:

1. Calculation based on effect size: the sample size is calculated (34) as $n=\frac{{\left(Z\,\alpha_{/2}+Z_{\beta }\right)}^{2}\times k}{f^{2}}$. To ensure adequate power (90%) for detecting significant differences in acoustic features across groups (dysphagia vs. non-dysphagia; and across the three audio modalities) with a small effect size (*f* = 0.1), a minimum of 7,288 audio samples was required, accounting for a 15% attrition rate due to quality control.
2. Calculation based on machine learning requirements: adhering to the widely accepted heuristic for binary classification models, we ensured that the number of outcome events would be at least 10 times the number of candidate predictor variables (*n* = 23), requiring a minimum of 230 audio samples with a positive outcome.
3. Translation to participant number: the most stringent requirement came from the effect size calculation (7,288 samples). Given that each participant would contribute a minimum of 12 audio samples, the required number of participants was 608.

### 2.2 Outcome variables and assessment tools

The binary outcome variable was defined based on the Water Swallow Test (WST), a practical and widely used screening tool that, while imperfect, provides a standardized reference for initial risk assessment. Audio data from participants with WST grades 3–5 were labeled as “high risk for dysphagia.”

### 2.3 Identify variables and evaluation tools

Based on literature evidence and prior validation, this study selected 23 discriminative acoustic features across four domains as model inputs: (1) five time-domain features (duration, mean amplitude, amplitude SD, skewness, kurtosis); (2) 10 frequency-domain features (fundamental frequency, formants F1-F3, peak/center frequencies, bandwidth, mean/SD harmonic-to-noise ratio, spectral centroid); (3) three energy features (total energy, average/peak power); and (4) five non-linear features (amplitude/frequency perturbation, fractal dimension, aggregation/average entropy).

### 2.4 Acoustic data acquisition

**FIGURE 2:**
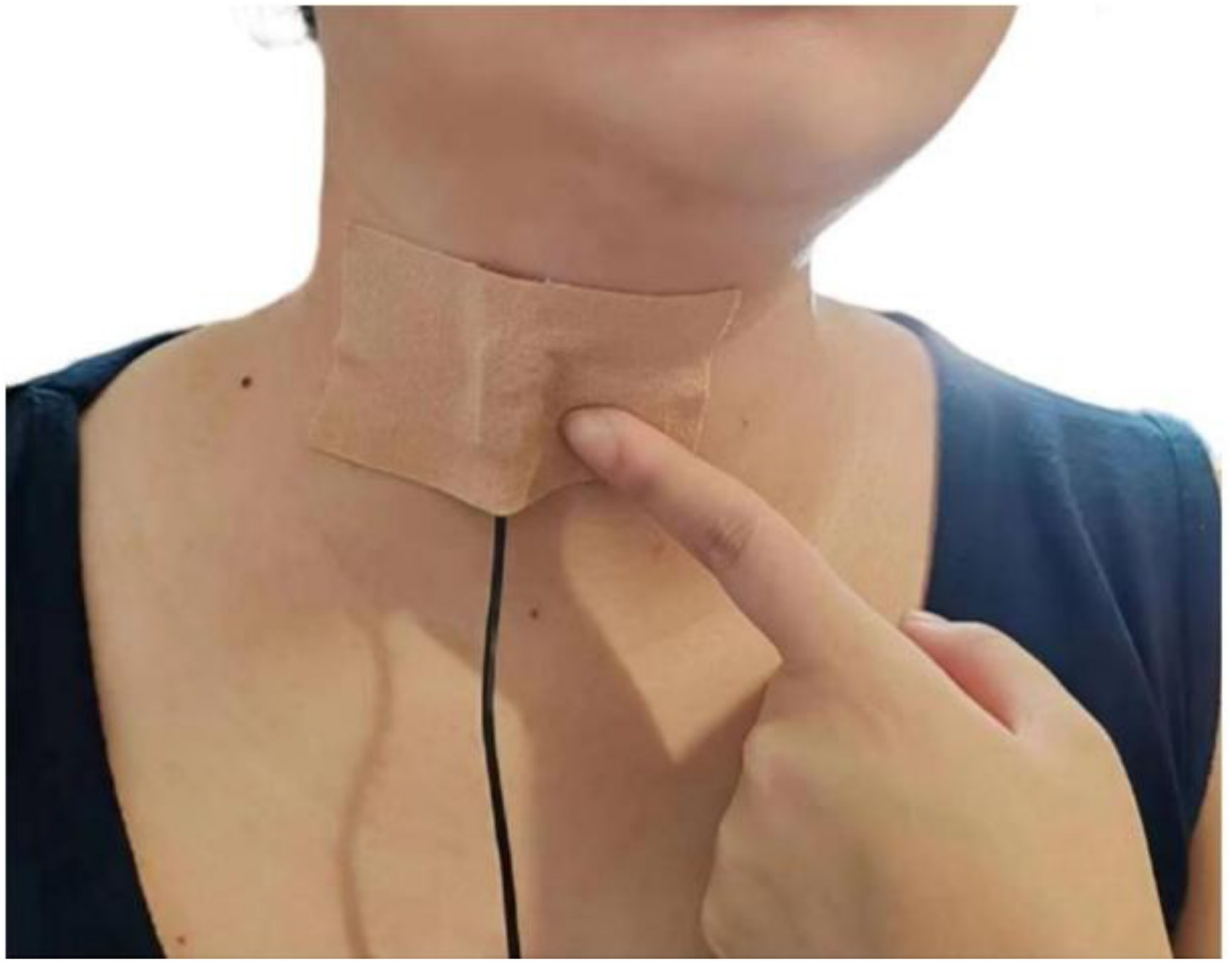
Microphone placement and securement.

All audio was recorded on-site in isolated rooms within the nursing homes to ensure acoustic fidelity. We used a contact microphone (C411L, AKG) affixed inferior to the cricoid cartilage (35, 36) (Figure 2) and connected to a digital recorder (Portacapture X8, TASCAM) configured at a 44.1 kHz sampling rate and 24-bit depth. Participants were positioned in a standardized seated posture with their backs fully supported against the chair backrest, feet flat on the floor, and knees flexed at approximately 90 degrees to ensure stability. The head and neck were maintained in a neutral position throughout recording, and participants were instructed to avoid head movements to maintain consistent microphone placement and signal integrity. The recording protocol included: (1) A 10-s baseline ambient noise capture; (2) A series of predefined vocal, swallowing, and cough tasks performed sequentially (detailed in Supplementary Table 1); (3) A concluding 10-s ambient recording.

### 2.5 Dataset construction

The modeling dataset comprised 4,965 high-quality audio samples from 419 participants after quality control (see Supplementary Figure 1 for the detailed construction process). For external validation, a refined dataset of 2,957 samples from 216 subjects was prospectively collected using identical protocols (Supplementary Figure 2).

### 2.6 Model predictors

From the initial 23 extracted acoustic features in the training set, LASSO regression (37, 38) identified 12 discriminative variables for elderly dysphagia recognition: (1) two time-domain features (audio duration, skewness); (2) four frequency-domain features (center frequency, formants F1–F2, frequency bandwidth); (3) two energy-related features (total energy, peak power); and (4) four non-linear features (amplitude perturbation, waveform fractal dimension, aggregation entropy, average entropy).

### 2.7 Statistics

The determination of an adequate minimum sample size for developing a multivariate predictive model hinges on ensuring a robust representation of individuals and outcome events in relation to predictor parameters (39). The combined sample size from Beijing’s eight nursing homes and Shijiazhuang’s five nursing homes provided ample support for utilizing 12 predictive variables in both modeling and validation phases. Specifically, there were 419 cases (4,965 audio samples) in the modeling dataset, encompassing 104 (24.82%) patients who experienced dysphagia; while external validation involved 216 cases (2,957 audio samples), encompassing 62 (28.0%) patients who experienced dysphagia. Statistical analyses were performed according to the data characteristics. Normally distributed continuous variables were expressed as mean ± standard deviation and compared using Student’s *t*-tests. Non-normally distributed variables were reported as median (interquartile range) with between-group comparisons analyzed by Mann-Whitney *U*-tests. Categorical data were presented as counts and percentages, with chi-square tests used for group comparisons. Statistical significance was defined as *p* < 0.05 for all analyses. All the statistical analyses of this study were conducted in the Python3.12 software environment.

### 2.8 Model development and comparison

The modeling dataset was randomly split into a training set (80%) and an internal test set (20%) for model development and initial validation. To address class imbalance, the Synthetic Minority Oversampling Technique (SMOTE) was applied to the training data. Using the 12 predictor variables selected by LASSO regression, we constructed and compared four distinct machine learning algorithms: Logistic Regression, Random Forest, Support Vector Machine (SVM), and XGBoost. Model performance was evaluated based on discrimination (assessed by the area under the receiver operating characteristic curve, AUC), calibration, and clinical utility (using decision curve analysis). The optimal model was subsequently interpreted using SHapley Additive exPlanations (SHAP) to quantify feature contributions and was finally validated on the independent external validation set.

All analyses were conducted in Python 3.12 using standard libraries (scikit-learn, XGBoost). Model hyperparameters were optimized via cross-validation (detailed configurations are provided in Supplementary Table 2).

### 2.9 Ethics

This study received ethical approval (IRB00001052-23160). Written informed consent was obtained from both institutional authorities (participating nursing homes and community health service stations) and individual participants prior to data collection. All collected survey data were maintained with strict confidentiality, and the entire research process complied with ethical requirements.

## 3 Results

### 3.1 Characteristics of the model development set

A total of 419 participants were included in the model development cohort. Based on the Water Swallow Test, 104 participants (24.82%) were identified as having dysphagia (WST grade ≥ 3). Comparative analysis revealed that the dysphagia and non-dysphagia groups showed statistically significant differences (*P* < 0.05) in key clinical characteristics, including the number of medication types, history of dysphagia-related diseases, BMI, nutritional status, daily activity ability, and MMSE scores. Detailed demographic and clinical characteristics for the entire cohort and by group are provided in Supplementary Table 3.

The 4,965 high-quality audio samples formed the basis for feature extraction. A total of 23 acoustic features were extracted from each sample, which were then combined with the outcome variable to create the final modeling dataset. A detailed description of the extracted acoustic features is provided in Supplementary Table 4.

### 3.2 Characteristics of the external validation set

The external validation cohort consisted of 216 participants. Among them, 62 (28.70%) were classified as having dysphagia (WST grade ≥ 3), which is comparable to the prevalence in the development cohort. The basic characteristics of this cohort are summarized in Supplementary Table 5.

The external validation set consisted of 2,957 validated audio samples obtained from 216 participants. Complete data specifications and categorical distributions are detailed in Supplementary Table 6.

### 3.3 Variable screening

LASSO regression identified 12 predictive acoustic features from the initial set of 23. The selected features encompassed temporal, spectral, energy, and non-linear domains, including audio duration, formants F1 and F2, and fractal dimension (a complete list is provided in see section “2.6 Model predictors”).

Analysis of the feature selection process revealed that formant F1, fractal dimension, and aggregation entropy were the most robust predictors, as they maintained non-zero coefficients across the strongest regularization penalties (Supplementary Figure 3). The optimal regularization parameter was determined via 10-fold cross-validation (Supplementary Figure 4).

This refined set of 12 features was used for all subsequent model development, following appropriate data balancing.

### 3.4 Interpretation of the model

**FIGURE 3:**
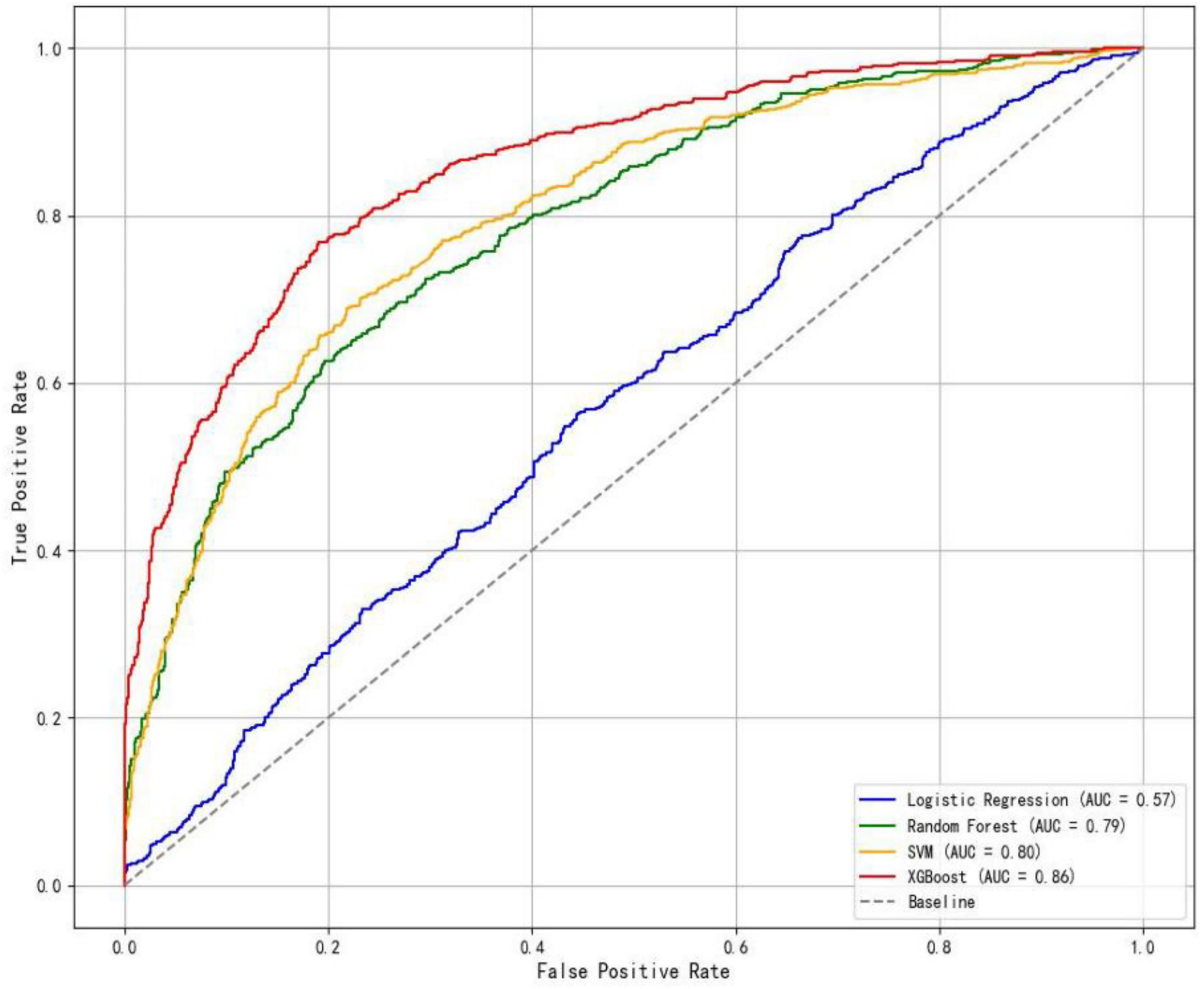
ROC curves of the four models.

**FIGURE 4:**
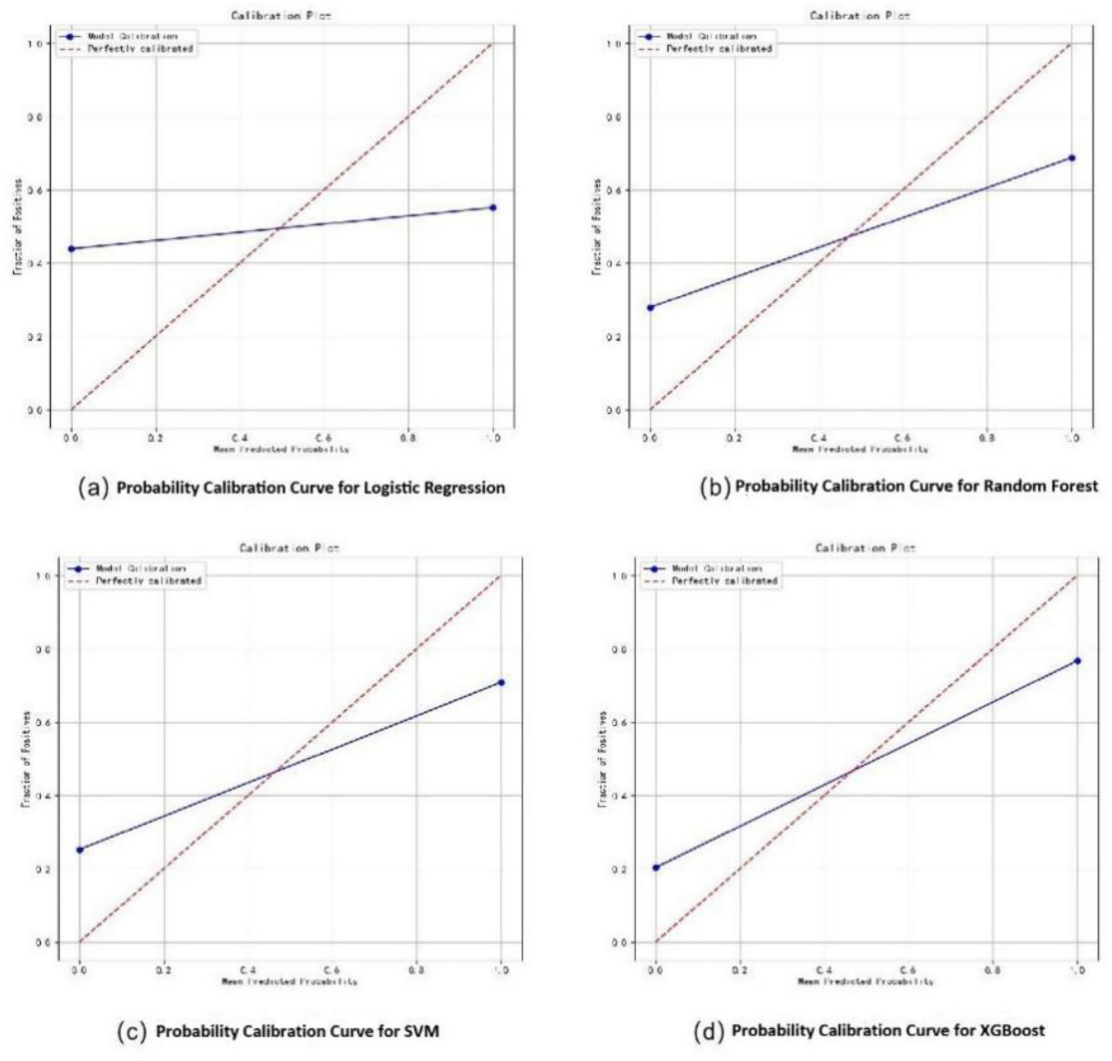
Probability calibration curves for dysphagia recognition models, including Logistic Regression **(a)**, Random Forest **(b)**, SVM **(c)**, and XGBoost **(d)**.

**FIGURE 5:**
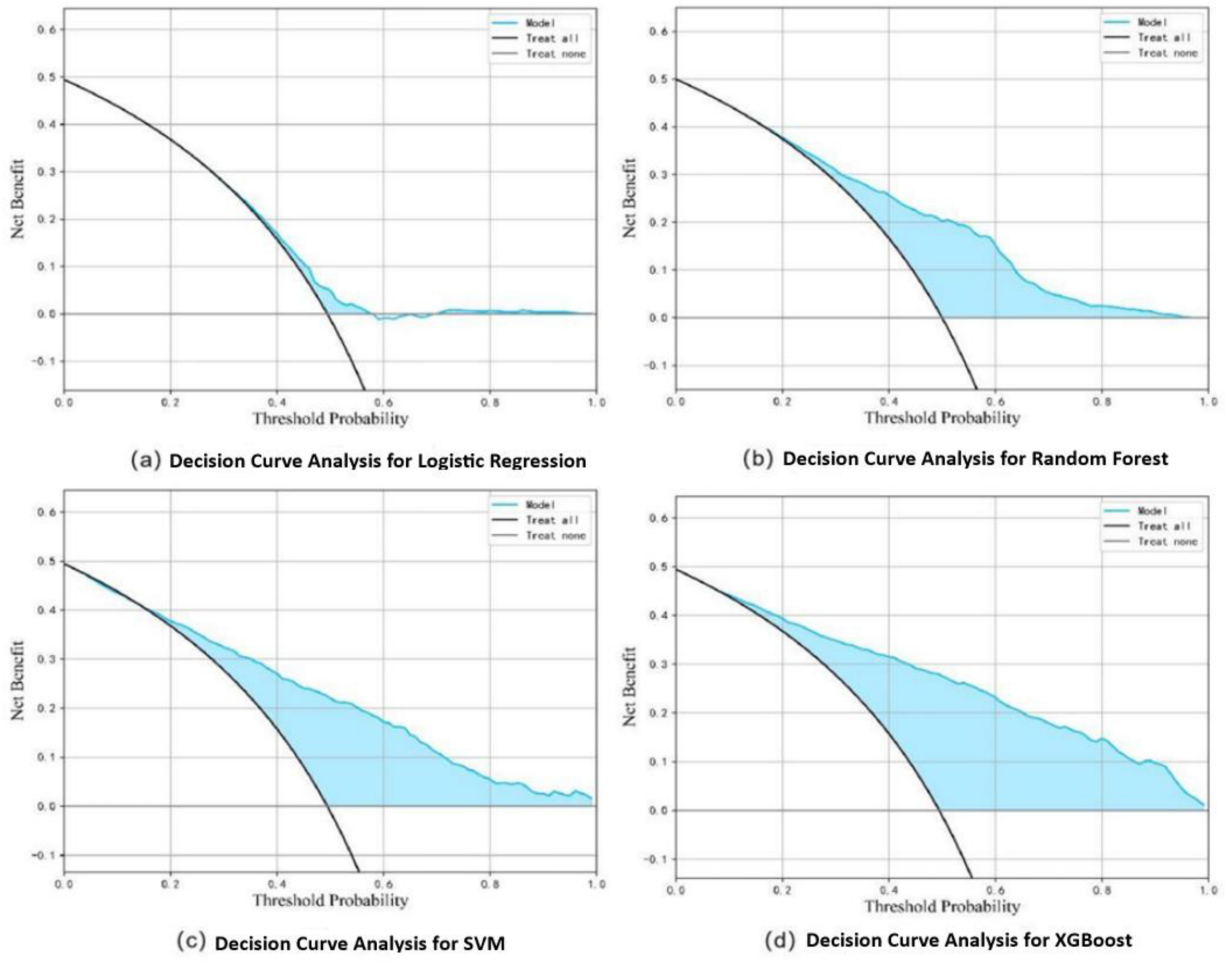
Decision curve analysis for dysphagia recognition models, including Logistic Regression **(a)**, Random Forest **(b)**, SVM **(c)**, and XGBoost **(d)**.

In the training set, the XGBoost model exhibited the highest accuracy at 0.78, significantly outperforming both Random Forest (0.70) and SVM (0.73) (*P* < 0.05). In terms of AUC value comparison, the XGBoost model achieved a value of 0.86 (95% CI: 0.81∼0.91), significantly outperforming other models (*P* < 0.05). And the XGBoost model demonstrated optimal sensitivity (0.80), correctly identifying 80% of elderly dysphagia cases, while maintaining superior specificity (0.76). The probability calibration curve of the XGBoost model is closest to the ideal calibration line and shows good calibration effects in different probability intervals. In terms of clinical efficacy, the XGBoost model shows the highest net benefit in the decision curve analysis, especially in the medium and high threshold range (0.3–1.0), and can provide the maximum benefit for clinical decision-making (Figures 3–5). The comprehensive comparison of all performance metrics is presented in Supplementary Table 7.

### 3.5 Internal and external validation of the final model

Internal validation of the XGBoost model on the hold-out test set demonstrated an AUC of 0.86 (95% CI: 0.81–0.91), with an accuracy of 0.78, a sensitivity of 0.80, and a specificity of 0.76.

External validation revealed that the model’s performance when applied directly to the independent cohort was initially limited (AUC = 0.52). Following hyperparameter optimization on the external validation set, the model’s discrimination improved to an AUC of 0.71 (95% CI: 0.67–0.75) and showed good calibration.

The ROC curves, calibration plots, and feature importance rankings for the final model in both internal and external validations are provided in Supplementary Figures 5–8.

## 4 Discussion

This study has developed a ML model based on EMR data from multiple center hospitals, demonstrating strong discriminative ability and clinical utility in identifying dysphagia in the elderly. The model’s performance has been externally validated across regions. Compared with traditional assessment methods such as the Water Swallow Test, it has the following advantages: (1) Objective acoustic analysis can reduce the deviation of human assessment; (2) It is easy to operate and suitable for community screening; (3) Combined with intelligent devices, it is expected to achieve remote monitoring and provide emergency early warnings such as aspiration and asphyxia. The model demonstrates significant clinical value by effectively assisting healthcare providers in identifying high-risk dysphagia patients, particularly in resource-limited settings. Its implementation facilitates early intervention, reduces complication rates, and advances timely diagnosis and treatment for elderly populations in China.

This study presents a novel approach to dysphagia screening by developing a machine learning model based on multimodal acoustic features specifically for community-dwelling the elderly. Unlike previous studies that often relied on single-modality analysis or focused on neurologically impaired populations, our model leverages the integration of voice, cough, and swallow sounds to capture a more comprehensive picture of swallowing physiology in a broader geriatric population. The rigorous external validation across different geographic regions further underscores the model’s generalizability and practical application potential for large-scale community screening.

It is widely acknowledged that ML models offer superior predictive performance compared to traditional linear models (40). This advantage enables the construction of a relatively robust model from complex data (41). ML excels in handling heterogeneous multidimensional data, as demonstrated by the inclusion of 12 predictive variables in this study, such as the audio duration and skewness, which exhibited high heterogeneity.

Recent studies have focused on the accuracy of identifying dysphagia. Donohue et al. (42) used a linear hybrid model and a machine learning classifier to distinguish the swallowing ability of healthy individuals and patients with neurodegenerative diseases based on 170 swallowing data from 20 patients with neurodegenerative diseases and 171 swallowing data from 51 healthy adults. Although they achieved a relatively high accuracy rate (99%), the sample size was limited and there was a lack of cross-population validation. Steele et al. (43) developed an LDA model based on samples from 305 patients with oropharyngeal dysphagia from seven medical centers in the United States. During the swallowing of liquid barium, VFSS and biaxial acceleration signals were simultaneously collected, achieving an average AUC of 81.5%. The advantage of this model lies in the use of multi-center data, which enhanced the representativeness of the model. This study, through large-sample cross-regional external validation, not only surpassed the research of Donohue et al. (42) in sample size, but also outperformed the results of Steele et al. (43) in classification performance (AUC = 0.86). These findings not only confirm the theoretical validity of the model, but also highlight its reliability and universality in clinical application through strict cross-regional validation, effectively compensating for the methodological limitations of existing studies that emphasize model performance over practical application value.

We developed and externally validated a screening model for dysphagia in the elderly using 12 acoustic features derived from participants across 13 nursing homes in Beijing and Shijiazhuang. Ultimately, the XGBoost algorithm yielded a model with superior accuracy (0.78), AUC (0.86), and clinical net benefit performs the best, especially in the medium and high threshold range (0.3–1.0). Similarly, this model demonstrated excellent performance on both internal and external validation sets.

Furthermore, in this study, by integrating multimodal acoustic characteristics, while maintaining a comparable accuracy rate to the single-modal study, it maintained a relatively high sensitivity (0.84) and specificity (0.79), achieving a balance between sensitivity and specificity. At the same time, the misdiagnosis rate (false positive) could be controlled below 21%, and the missed diagnosis rate (false negative) could be controlled within 16%.

A critical consideration is the fundamental advantage of this acoustic model over simpler, established screening tools like the EAT-10 questionnaire (44). While the EAT-10 relies on subjective patient self-reporting, which can be unreliable in older adults with cognitive impairment or lack of insight, our model provides a fully objective and quantitative assessment. It does not depend on patient comprehension or subjective symptom reporting. The acoustic analysis automates the screening process, reducing reliance on clinical intuition and potential bias in human-administered swallowing trials. Therefore, its primary advantage lies in its potential to offer a standardized, objective, and automatable first-line screening method, particularly valuable in settings with high patient volume or limited specialist availability, and for populations where traditional self-reporting tools are less effective.

This study has several limitations that should be acknowledged. The primary limitation is the use of the Water Swallow Test (WST) as the reference standard instead of instrumental assessments like videofluoroscopy (VFSS) or endoscopy (FEES). This pragmatic choice was necessitated by the large-scale, community-based design of our study. Consequently, our model should be interpreted as a screening tool for dysphagia risk, not a definitive diagnostic instrument. The model’s performance is inherently bounded by the accuracy of the WST, particularly in detecting silent aspiration. Future research must validate this model against VFSS/FEES in a clinical setting to establish its diagnostic accuracy and ability to detect silent aspiration.

Additionally, at the data level, collecting multiple audio samples from each participant poses a risk of reducing data independence. Future larger-scale studies can adopt mixed-effects models for in-depth analysis. At the hardware level, the current equipment may cause discomfort during prolonged use due to rigid materials, and wired connections limit patient mobility. The lack of integrated remote, real-time data transmission also curtails its potential for telemedicine. Future iterations should focus on developing ergonomic, wireless devices to enhance user comfort and enable continuous monitoring.

In conclusion, we have developed a recognition model for dysphagia in the elderly based on acoustic features, which has clinical advantages and high promotion value. This is of great significance for improving the quality of life of patients, reducing medical costs caused by late diagnosis, and achieving early intervention and optimized management of dysphagia.

## Data Availability

The raw data supporting the conclusions of this article will be made available by the authors, without undue reservation.
