# Supplementary material for: Development and validation of a screening model for dysphagia in the elderly based on acoustic features
Source: Front Med (Lausanne). 2025 Dec 8;12:1719174. doi: 10.3389/fmed.2025.1719174 (PMC12722897; doi:10.3389/fmed.2025.1719174)
Supplement: Supplementary file 1 [file Supplementary_file_1.docx]

**Supplementary Materials**


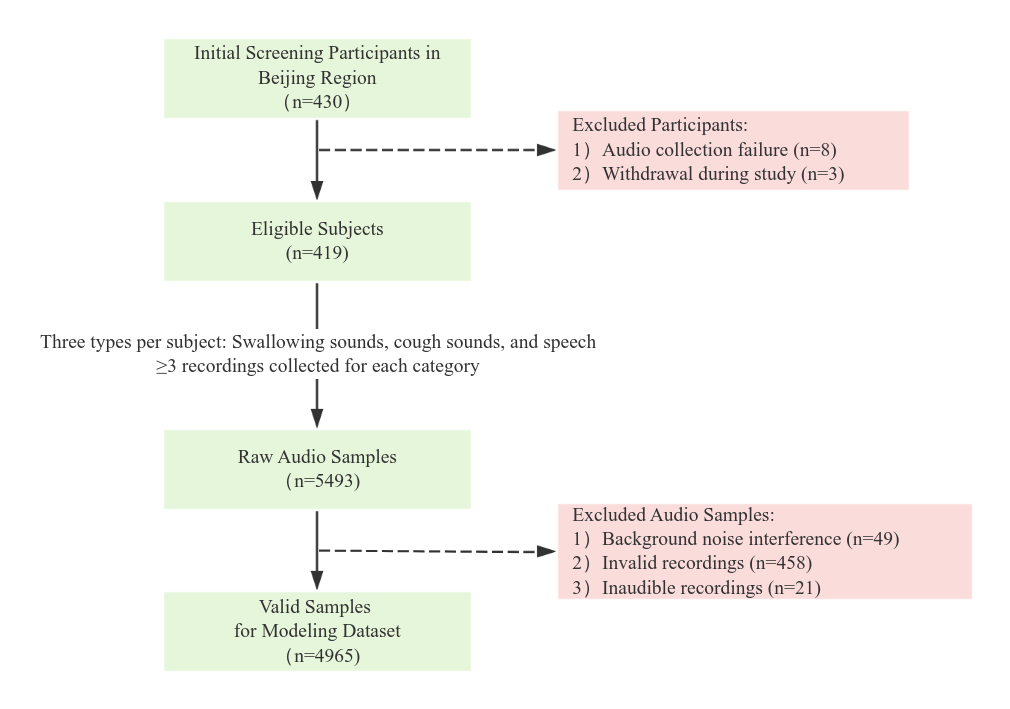


**Figure S1 Formation process of the modeling dataset**


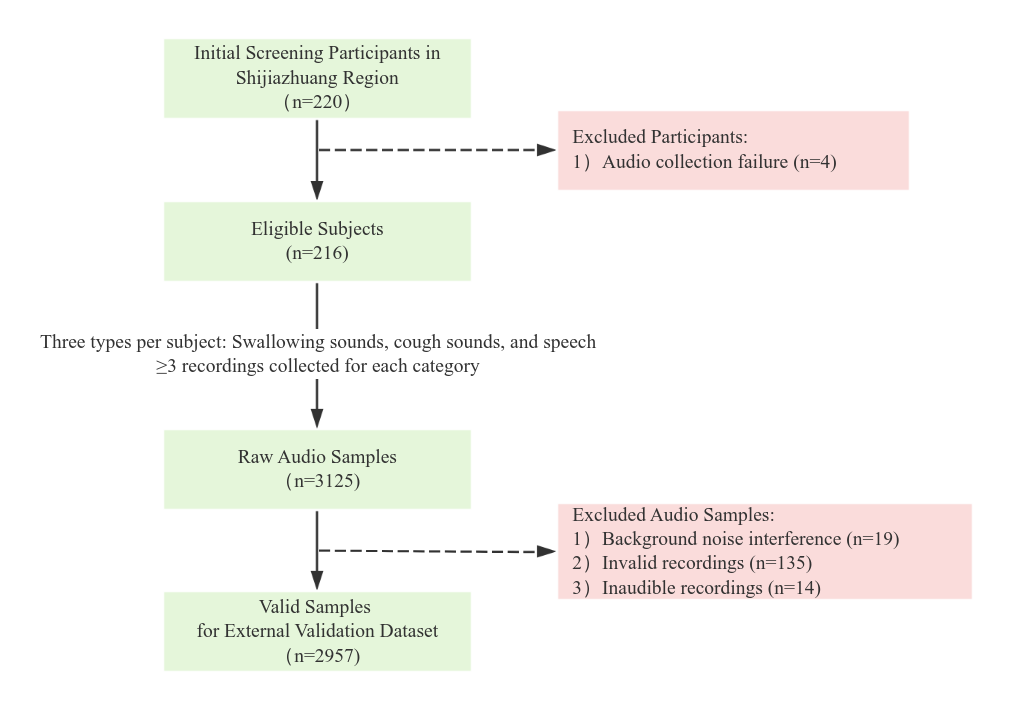


**Figure S2 Formation process of the external validation dataset**


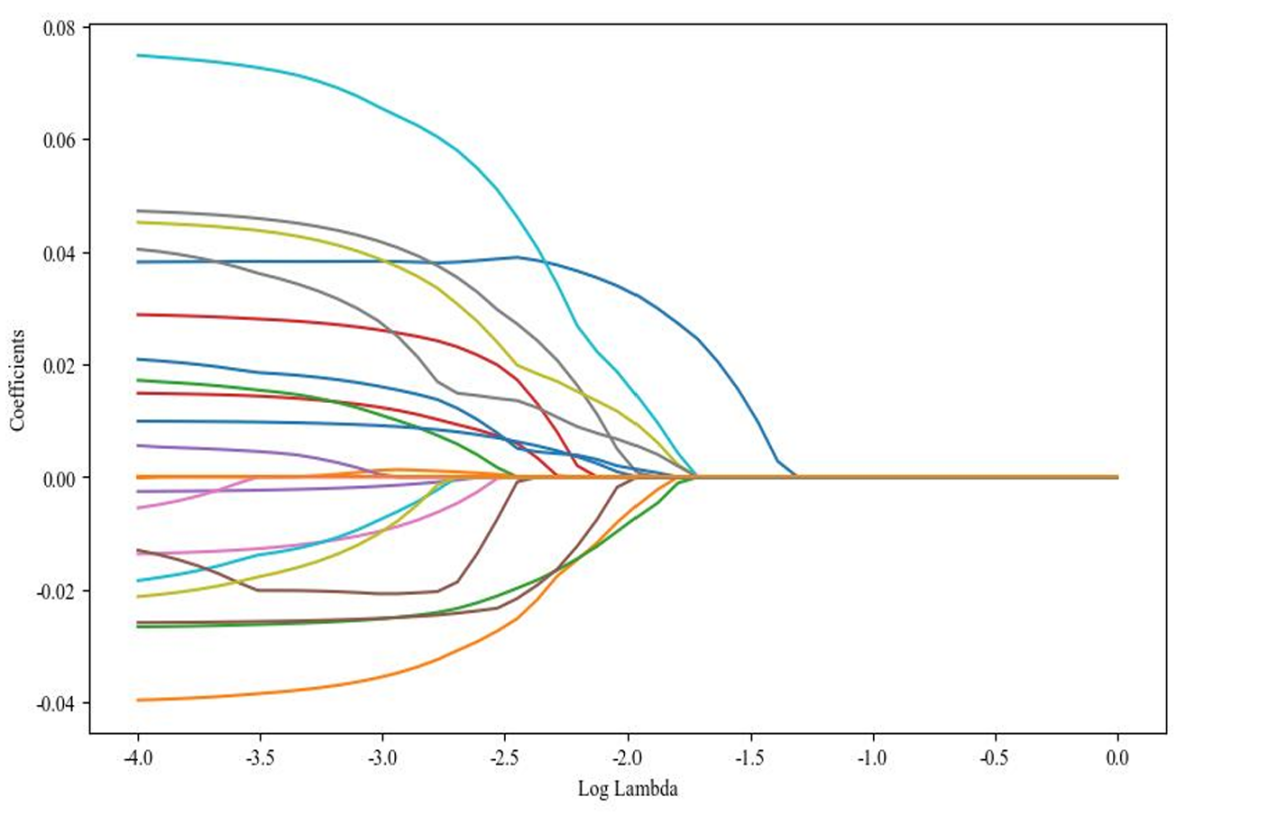


**Figure S3 Coefficient path of LASSO regression variables**


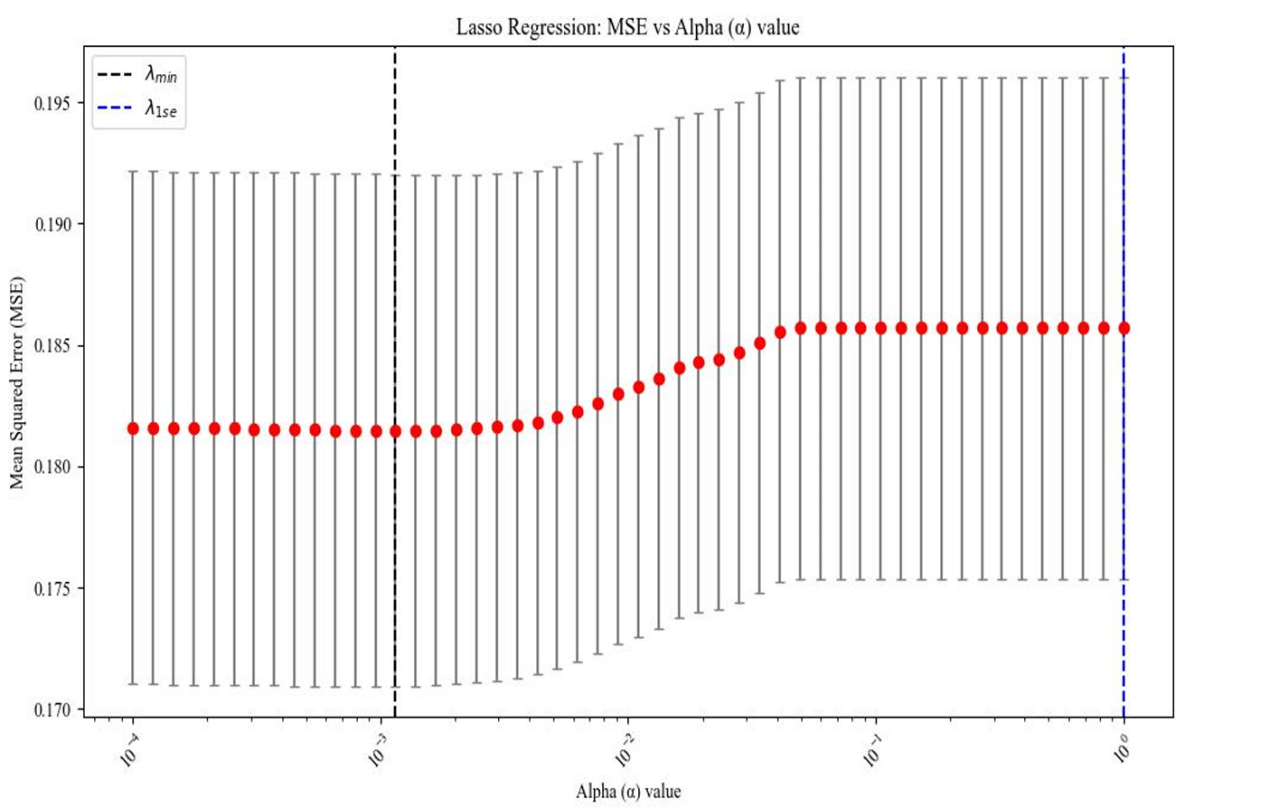


**Figure S4 Relationship between LASSO regularization parameter λ and mean squared error**


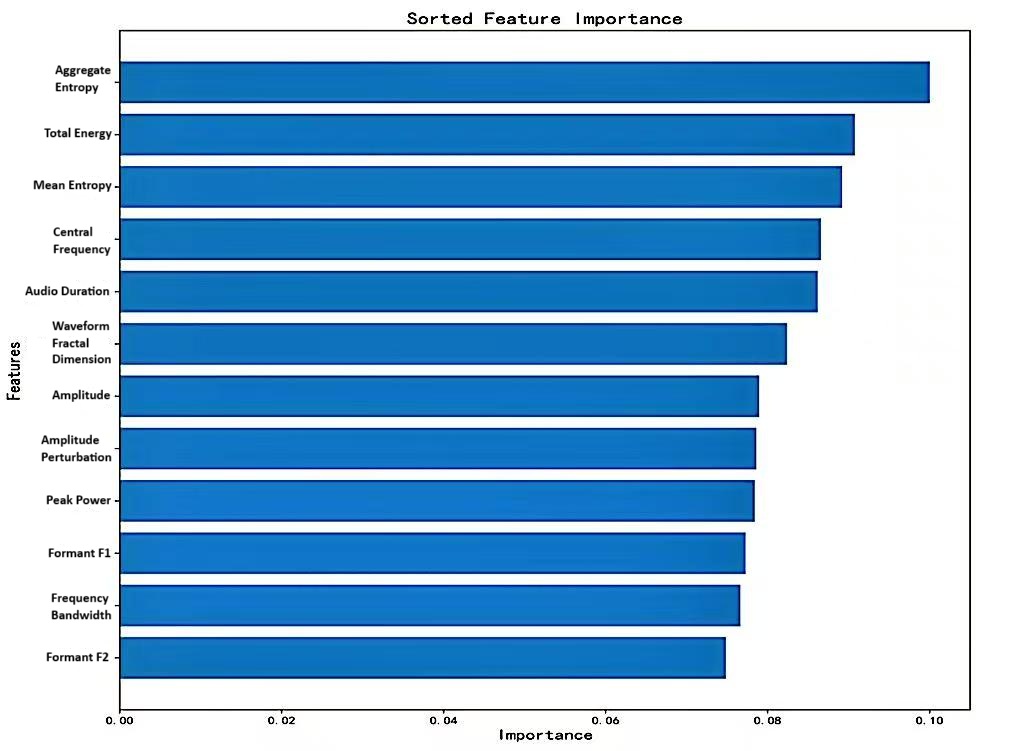


**Figure S5 Feature importance ranking of the optimal recognition model**


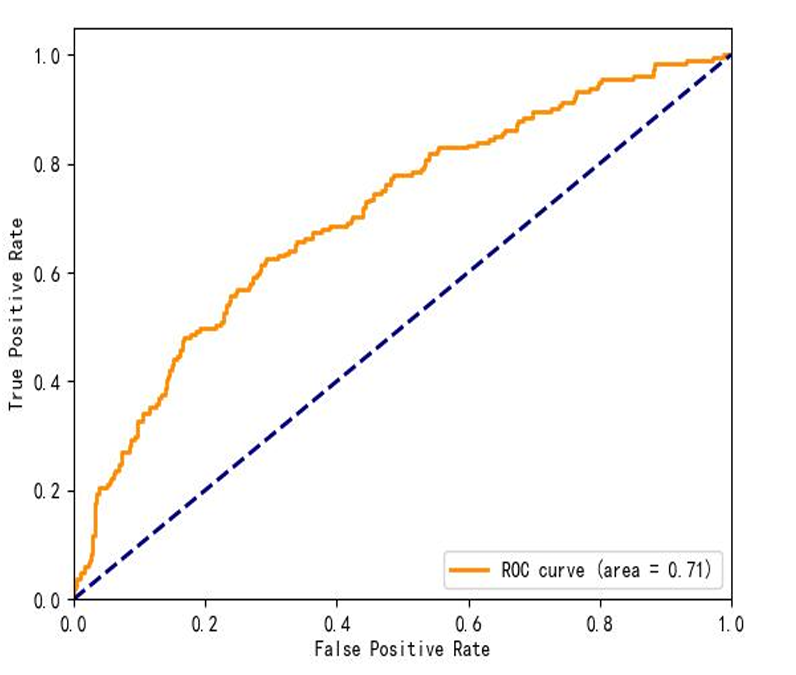


**Figure S6 ROC curve for the adjusted XGBoost model**


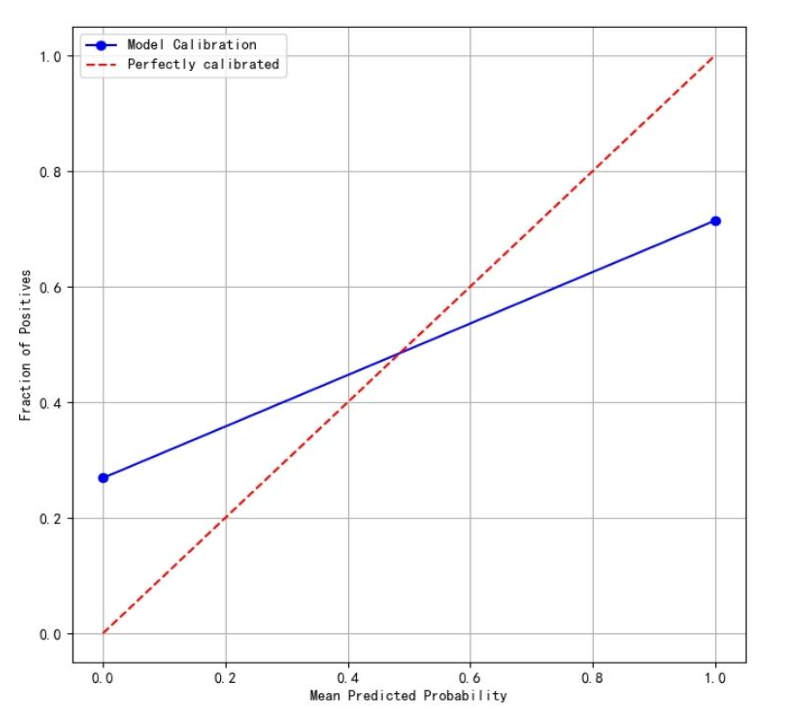


**Figure S7 Probability calibration curve of the adjusted XGBoost model**


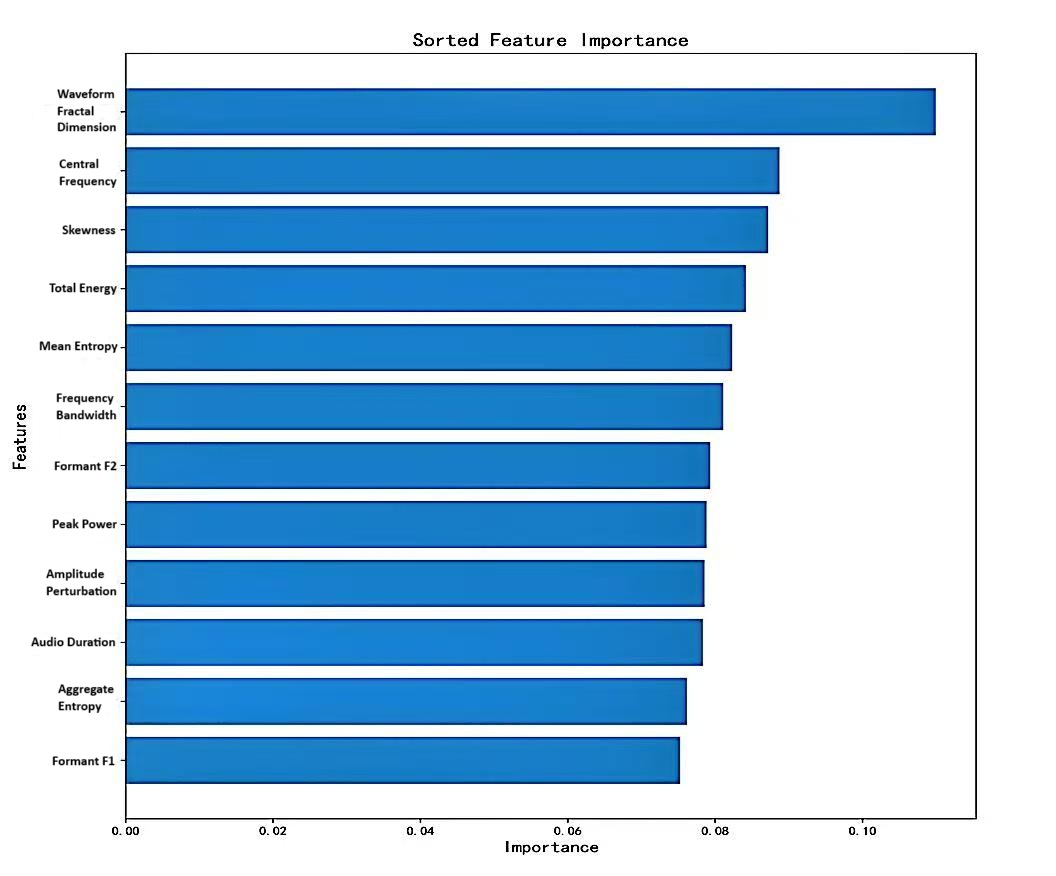


**Figure S8 Feature importance ranking of the calibrated XGBoost model**

**Table S1 Dynamic Acquisition Tasks**

| Step | Type | Task | Repetitions | Duration |
| --- | --- | --- | --- | --- |
| 1 | Voice | Phonating /a:/ | 3 | 2s |
| 2 | Cough | Voluntary Cough | 3 | Natural |
| 3 | Swallowing | Swallowing 10ml Water | 3 | Natural |
| 4 | Voice | Phonating /a:/ | 3 | 2s |

**Table S2 The implementation method and hyperparameter setting**

**of dysphagia recognition model in python**

| Model | Library/Tool | Hyperparameter Settings and Descriptions |
| --- | --- | --- |
| Logistic Regression | Scikit-learn | c: regularization parameter |
|  |  | solver: optimization algorithm |
| Random Forest | Scikit-learn | n_estimators: number of trees |
|  |  | max_depth: maximum tree depth |
|  |  | min_samples_leaf: minimum samples at leaf node |
|  |  | min_samples_split: minimum samples to split node |
|  |  | max_features: maximum number of features |
| SVM | Scikit-learn | c: penalty parameter |
|  |  | kernel: kernel type |
|  |  | gamma: kernel coefficient |
| XGBoost | xgboost | n_estimators: number of trees |
|  |  | max_depth: maximum tree depth |
|  |  | learning_rate: boosting learning rate |

**Table S3 Basic characteristics of the model development set (n=419)**

| Variable | Total (n=419) | No Dysphagia (n=315) | Dysphagia (n=104) | t/χ² value | P-value |
| --- | --- | --- | --- | --- | --- |
| Age (years) | 80.67±9.20 | 80.40±9.32 | 81.47±8.85 | -1.029 | 0.304 |
| Gender [n (%)] |  |  |  |  |  |
| Male | 185 (44.15) | 137 (43.49) | 48 (46.15) | 0.225 | 0.636 |
| Female | 234 (55.85) | 178 (56.51) | 56 (53.85) |  |  |
| Education [n (%)] |  |  |  |  |  |
| Illiterate | 63 (15.04) | 49 (15.56) | 14 (13.46) | 0.470 | 0.925 |
| Primary school | 72 (17.18) | 53 (16.83) | 19 (18.27) |  |  |
| Junior/High school | 201 (47.97) | 152 (48.25) | 49 (47.12) |  |  |
| College or above | 83 (19.81) | 61 (19.37) | 22 (21.15) |  |  |
| Marital status [n (%)] |  |  |  |  |  |
| Married | 138 (32.94) | 107 (33.97) | 31 (29.81) | 0.613 | 0.434 |
| Unmarried/Divorced/Widowed | 281 (67.06) | 208 (66.03) | 73 (70.19) |  |  |
| Living arrangement [n (%)] |  |  |  |  |  |
| Living alone | 312 (74.46) | 240 (76.19) | 72 (69.23) | 1.992 | 0.158 |
| Not living alone | 107 (25.54) | 75 (23.81) | 32 (30.77) |  |  |
| Household income per capita (¥/month) |  |  |  |  |  |
| <2000 | 31 (7.40) | 24 (7.62) | 7 (6.73) | 0.190 | 0.909 |
| 2000-3000 | 64 (15.27) | 47 (14.92) | 17 (16.35) |  |  |
| >3000 | 324 (77.33) | 244 (77.46) | 80 (76.92) |  |  |
| History of dysphagia-related diseases [n (%)] |  |  |  |  |  |
| Yes | 194 (46.30) | 179 (56.83) | 46 (44.23) | 4.988* | 0.026 |
| No | 225 (53.70) | 136 (43.17) | 58 (55.77) |  |  |
| History of head/neck surgery/treatment [n (%)] |  |  |  |  |  |
| Yes | 42 (10.02) | 26 (8.25) | 16 (15.38) | 4.408 | 0.036 |
| No | 377 (89.98) | 289 (91.75) | 88 (84.62) |  |  |
| Number of chronic diseases | 2.11±1.27 | 2.07±1.28 | 2.23±1.24 | -1.145 | 0.253 |
| Number of medications | 1.50±0.98 | 1.43±0.96 | 1.71±1.00 | -2.550 | 0.011 |
| BMI (kg/m²) | 24.87±3.67 | 25.08±3.53 | 24.22±4.00 | 2.099 | 0.036 |
| Nutritional status [n (%)] |  |  |  |  |  |
| Normal | 173 (41.29) | 144 (45.71) | 29 (27.88) | 21.186 | <0.001 |
| At risk | 202 (48.21) | 149 (47.30) | 53 (50.96) |  |  |
| Malnourished | 44 (10.50) | 22 (6.98) | 22 (21.15) |  |  |
| ADL score (points) | 69.40±30.08 | 74.92±26.42 | 52.69±34.18 | 6.888 | <0.001 |
| MMSE score (points) | 17.83±7.11 | 18.50±7.07 | 15.79±6.86 | 3.417 | 0.001 |

**Table S4 Data description and classification in the model development set**

| Outcome Label | Kubota Water Swallow Test Grade | Subjects in Modeling Dataset (n) | Samples in Modeling Dataset (n) | Acoustic Features Extracted (n) |
| --- | --- | --- | --- | --- |
| Non-Dysphagia | Grade 1-2 | 315 | 3,741 | 86,043 |
| Dysphagia | Grade 3-5 | 104 | 1,224 | 28,152 |

**Table S5 Basic characteristics of the external validation dataset (n=216)**

| **Variable** | **Total (n=216)** | **Non-Dysphagia Group (n=154)** | **Dysphagia Group (n=62)** | | **t/χ² value** | **P-value** |
| --- | --- | --- | --- | --- | --- | --- |
| Age (years) | 81.59±8.75 | 80.63±8.71 | 83.97±8.43 | -2.570 | | 0.011 |
| Gender [n (%)] |  |  |  |  | |  |
| Male | 81 (37.50) | 53 (34.42) | 28 (45.16) | 2.178 | | 0.140 |
| Female | 135 (62.50) | 101 (65.58) | 34 (54.84) |  | |  |
| Education [n (%)] |  |  |  |  | |  |
| Illiterate | 14 (6.48) | 9 (5.84) | 5 (8.06) | 3.125 | | 0.373 |
| Primary school | 39 (18.06) | 28 (18.18) | 11 (17.74) |  | |  |
| Junior/High school | 93 (43.06) | 62 (40.26) | 31 (50.00) |  | |  |
| College or above | 70 (32.41) | 55 (35.71) | 15 (24.19) |  | |  |
| Marital status [n (%)] |  |  |  |  | |  |
| Married | 92 (42.59) | 68 (44.16) | 24 (38.71) | 0.536 | | 0.464 |
| Unmarried/Divorced/Widowed | 124 (57.41) | 86 (55.84) | 38 (61.29) |  | |  |
| Living arrangement [n (%)] |  |  |  |  | |  |
| Living alone | 89 (41.20) | 62 (40.26) | 27 (43.55) | 0.197 | | 0.657 |
| Not living alone | 127 (58.80) | 92 (59.74) | 35 (56.45) |  | |  |
| Household income per capita (¥/month) |  |  |  |  | |  |
| <2000 | 18 (8.33) | 12 (7.79) | 6 (9.68) | 0.743 | | 0.690 |
| 2000-3000 | 19 (8.80) | 15 (9.74) | 4 (6.45) |  | |  |
| >3000 | 179 (82.87) | 127 (82.47) | 52 (83.87) |  | |  |
| History of dysphagia-related diseases [n (%)] |  |  |  |  | |  |
| Yes | 119 (55.09) | 84 (54.55) | 35 (56.45) | 0.065 | | 0.799 |
| No | 97 (44.91) | 70 (45.45) | 27 (43.55) |  | |  |
| History of head/neck surgery/treatment [n (%)] |  |  |  |  | |  |
| Yes | 13 (6.02) | 11 (7.14) | 2 (3.23) | 1.199 | | 0.274 |
| No | 203 (93.98) | 143 (92.86) | 60 (96.77) |  | |  |
| Number of chronic diseases | 1.92±1.35 | 1.87±1.31 | 2.03±1.46 | -0.797 | | 0.426 |
| Number of medications | 4.38±3.28 | 4.31±3.16 | 4.53±3.56 | -0.447 | | 0.655 |
| BMI (kg/m²) | 23.23±4.04 | 23.05±3.81 | 23.68±4.55 | -0.967 | | 0.335 |
| Nutritional status [n (%)] |  |  |  | 3.064 | | 0.216 |
| Normal | 111 (51.39) | 83 (53.90) | 28 (45.16) |  | |  |
| At risk | 89 (41.20) | 58 (37.66) | 31 (50.00) |  | |  |
| Malnourished | 16 (7.41) | 13 (8.44) | 3 (4.84) |  | |  |
| ADL score (points) | 62.48±35.61 | 67.27±34.17 | 50.56±36.60 | 3.185 | | 0.002 |
| MMSE score (points) | 19.13±7.94 | 20.18±7.52 | 16.50±8.39 | 3.146 | | 0.002 |

**Table S6 Data description and classification in the external validation dataset**

| **Outcome Label** | **Kubota Water Swallow Test Grade** | **Participants in External Validation Set (n)** | **Audio Samples in External Validation Set (n)** | **Acoustic Features Extracted (n)** |
| --- | --- | --- | --- | --- |
| Non-Dysphagia | Grade 1-2 | 154 | 2,083 | 47,909 |
| Dysphagia | Grade 3-5 | 62 | 874 | 20,102 |

**Table S7 Discriminative performance of four models in the test set**

| Model | AUC | Accuracy | Sensitivity | Specificity |
| --- | --- | --- | --- | --- |
| Logistic Regression | 0.57 | 0.56 | 0.55 | 0.56 |
| Random Forest | 0.79 | 0.70 | 0.74 | 0.66 |
| SVM | 0.80 | 0.73 | 0.76 | 0.69 |
| XGBoost | 0.86 | 0.78 | 0.80 | 0.76 |
